# Supplementary material for: Are carbon emissions trading and green financial instruments synergistic? -Comprehensive quantitative research based on content analysis
Source: PLoS One. 2024 Mar 7;19(3):e0298601. doi: 10.1371/journal.pone.0298601 (PMC10919601; doi:10.1371/journal.pone.0298601)
Supplement: S1 Data — (ZIP) [file pone.0298601.s001.zip › Data/地级市Z_F工作报告.zip!/┴╔─■/┴╔╤⌠╩╨/└┤╫╘╣1⁄2╓┌║┼í╛┬φ┐╦╩2╛▌═°í┐_2017.docx]

各位代表：

　　我代表市政府向大会报告工作，请予审议，并请市政协委员和列席会议的同志提出意见。

　　一、2016年工作回顾

　　刚刚过去的2016年，经济形势错综复杂，下行压力持续加大，全市经济发展遇到了一系列新问题新挑战。一年来，在省委、省政府和市委的正确领导下，市政府团结带领全市人民，深入贯彻落实党的十八大和十八届三中、四中、五中、六中全会精神，认真学习贯彻习近平总书记系列重要讲话精神，坚持稳中求进工作总基调，着力推进供给侧结构性改革，全面开展“四个辽阳”建设，攻坚克难，拼搏进取，保持了经济平稳发展、民生持续改善、社会和谐稳定。

　　预计，地区生产总值实现928亿元，一般公共预算收入实现70.8亿元，固定资产投资实现197亿元，城乡常住居民人均可支配收入分别实现28110元和12880元。

　　一年来，我们重点抓了以下八项工作。

　　（一）深入开展“项目年”活动，切实发挥有效投资的关键作用

　　千方百计抓项目。围绕“十三五”规划和“四个辽阳”建设，精心谋划推进了230个亿元以上项目。扎实开展前期工作，建立审批绿色通道。全面实施市级领导包扶责任制，为企业解决土地、供电、供水等要素瓶颈，加速项目建设进程。建立考核、激励、约谈机制，严格奖惩问责，激发广大干部服务项目、企业加快发展的积极性和主动性。

　　多渠道筹措资金。积极开展政府和社会资本合作（PPP），引进社会资本57.5亿元，合作项目7个，数量列全省第1位。努力争取银行贷款，与国开行、农发行等金融机构签约融资额度500亿元。充分发挥国资公司的融资平台作用，为企业贷款担保9.9亿元，融资租赁10亿元。争取到位上级资金79.3亿元。推动企业直接融资69亿元。实现4家企业在“新三板”挂牌。

　　大力开展招商引资。组建市经济合作局，充实招商力量。创新招商方式，拓宽招商领域，组织产业链、点对点招商活动百余次，促成50个项目签约或开工建设。全年实际利用外资8390万美元，吸引国内实际到位资金28.5亿元。

　　亿元以上重点项目开（复）工123项，完成投资130亿元，竣工投产40项。“四个辽阳”重点工程项目建设进展顺利。

　　（二）全力以赴稳工业，促进产业结构优化升级

　　多措并举保企业。深入开展千名领导干部帮扶千户企业活动，促进136户停产半停产企业恢复生产。为27户企业解决“过桥”资金，新增中小微企业再贷款9亿元。实施“营改增”全覆盖，为企业让税5.4亿元。清理整顿涉企收费项目，为企业减负2.8亿元。落实直供电指标3.7亿度，为企业节约电费4500万元。盘活利用工业闲置厂房5万平方米。

　　推动产业迈向中高端。加快推进芳烃及精细化工产业基地建设，俄油加工改造项目正式开工，2万吨药用辅料一期竣工投产，16万吨天然脂肪醇项目全面达产。培育壮大铝合金精深加工产业基地，专用车厢、轿车车身焊装生产线、全铝家具和建筑模板项目投产或部分投产。做大做强装备制造及汽车零部件产业集群，新能源汽车变速箱、重柴高压共轨、航空密封件等项目开工建设，新能源汽车电机、电控及电驱动总成项目正式签约。7户企业智能化、数字化车间获批省试点示范项目，1户企业被评为省智能制造标杆企业。大力发展电子信息产业，3个项目竣工投产，5个项目签约。化解煤炭产能10万吨、钢铁产能392万吨，为产业发展腾出空间。

　　加快科技创新步伐。与中科院、大连理工等50多家科研院所建立紧密合作关系，建设7个产业技术创新联盟和技术平台，促成合作项目百余项。在德国、意大利筹建海外研发中心2个。新增国家地方联合工程研究中心1个、省级技术中心和研究中心8个。启动了“双百”企业培育工程。认定高新技术企业21户。成功获批国家知识产权质押融资试点市。

　　（三）加速建设国家现代农业示范区，努力提升农业农村发展水平

　　全力推进9个现代农业示范区（带）建设。实施了一批现代农业重点项目。弓长岭区、三禾农业园分别被评为国家级休闲农业示范区和五星级休闲农业示范点，西马峰镇被评为全国“一村一品”示范镇，形成了点线面结合、梯次推进的现代农业发展格局。

　　推动农业与二三产业融合发展。调减玉米种植面积8万亩。粮食种植面积197万亩，产量实现104万吨。流转土地10.2万亩，发展农民专业合作社127家、家庭农场100家。耕种收综合机械化率达到82%。培育壮大农产品加工业，实施了22个重点产业化项目，7个建成投产。加快农产品现代流通体系和肉菜流通追溯体系建设，建成农产品直销点121个，88家试点单位全部实现农产品质量可追溯管理，初步形成可复制的“辽阳模式”。全面启动辽阳县农村土地承包经营权抵押贷款试点，发放贷款45笔。灯塔市获批国家级一二三产业融合发展试点。连续14年无区域性重大动物疫情发生。

　　完善农业农村基础设施。建设高标准农田11.6万亩，灯塔市万亩水稻省级示范试点项目进展顺利。巩固提升农村饮水安全工程67处、惠及8.5万人，实施3个灌区节水改造工程，完成《城市防洪规划》修编工作，荣获省“大禹杯”竞赛先进单位称号。大中修、改扩建各级公路954公里。绿化村屯60个，建成美丽乡村示范村36个。

　　着眼于壮大县域经济和推进以人为核心的新型城镇化，在全省率先启动特色小镇建设。高标准编制完成温泉旅游、皮草购物等10个市级特色小镇建设规划。搭建融资平台，设立引导资金，实施了35个产业发展和基础设施重点项目。汤河镇晋升国家级特色小镇。佟二堡等5个特色乡镇通过省级专家评审。

　　大力开展精准脱贫。20个贫困村退出销号，10397名建档立卡贫困人口实现脱贫，家庭人均纯收入达到4015元，超过国家标准685元，没有一个孩子因贫辍学，家庭住房条件明显改善，大病住院医疗费用全部减免，实现“票子、孩子、房子、医疗”问题一起解决。

　　（四）突出产业特色，推动服务业发展提质增效

　　改造提升传统服务业。引导传统商场增加服务功能，3户大型零售企业实现向商业综合体转型。推进兴隆大家庭、万达广场等服务业重点项目建设，商业布局不断完善。加快改造传统市场，粮油、果品等4个专业市场搬迁工作积极推进。组织“夜经济”主题活动20余项、特色展会10余个，拉动消费持续增长。

　　加快发展生产性服务业。编制完成现代综合物流园区规划，积极破解土地、资金等瓶颈制约，园区重点项目建设有序推进。佟二堡保税仓库投入运营，2家国际皮草拍卖行正式入驻，实现交易额1亿美元。建成电子商务产业园和河东电商基地，入驻企业60余家，重点电商平台网络交易额突破18亿元。成功引进4家外埠金融机构，完成农商行全域化改革，总部银行已达4家。金融机构本外币各项存款余额突破2000亿元；新增贷款增长14.3%，居全省前列。

　　进一步壮大生活性服务业。加强旅游基础设施和景区建设，西土线旅游迎宾大道全线竣工，假日热带雨霖馆投入运营，太子岛建设初具形象。弓长岭区入选第二批“国家全域旅游示范区”创建名单，龙石风景区跻身省级生态旅游示范区。旅游总收入实现180亿元，增长13.7%。建成智慧养老信息平台，公办和民营养老机构新增床位3000张，获批国家级医养结合试点城市。成功举办首届国际滑雪赛、车模邀请赛、龙崛国际格斗系列赛，提升了体育发展水平。

　　（五）加强城市建设和管理，持续改善人居环境

　　提升河东新城服务功能。调整完善新城管理体制，开发建设和管理服务更加协调。襄平大桥正式通车，核心区和北部路网进一步拓展，建成河东变电站，基础设施不断完善。欢乐海水上乐园、居然之家开业运营，新城医院投入使用，新增商业网点300余家，新城人气正在加速集聚。

　　加快推进老城区改建。实施棚改项目38个，安置居民1.2万户。出台22条房地产去库存的优惠政策，成功举办春秋两季房交会，化解存量房源130万平方米。综合改造老旧小区20个，维修“弃管楼”371栋，市民居住环境不断改善。

　　完善城市基础设施。大型人防公交枢纽工程正式启动，北环路开工建设，河东交通综合枢纽一期、朝光和下王家公交首末站建成使用，西环路和西部路网前期工作进展顺利。维修改造胜利路等29条城市道路，美化、绿化31条道路。建成南环街街边游园、中韩友谊园，完成博爱园改造工程。燃气、供水等基础设施建设不断加强。环卫机械化清扫率达到80%，城市环境更加清洁。

　　加强城市管理。加快构建“大城管”格局，建成数字化城市管理信息系统。因地制宜综合“治堵”，渠化调整主要路段和交通节点，开展道路“交通乱象”专项整治，有效缓解了交通压力。顺应群众需求，规范市场管理。开展再生资源回收站点、违章建筑、露天烧烤等整治行动，城市形象明显改观。

　　改善生态环境。深入开展环保违规项目清理整顿，淘汰关闭违规项目295个。治理闭坑矿山，荒山造林、森林抚育7000亩，重点林区基本实现全域封禁。加强水污染治理，完成2个污水处理厂改造，主要污染物排放持续降低。开展大气污染专项整治，购置新能源公交车40台，安装节能环保炉具4800台，全面禁烧秸秆，超额完成城区燃煤锅炉拆除和黄标车淘汰任务，空气质量优良天数比例进一步提升。

　　（六）深化改革开放，不断完善体制机制

　　推进“放管服”三管齐下，加快转变政府职能。持续加大简政放权力度，调整了173项行政职权，全部纳入清单管理。在全省率先启动相对集中行政审批权改革，成立市行政审批局，实行了“一枚公章”管审批的工作模式。积极推行“双随机、一公开”监管机制，覆盖市场监管执法事项70%、其他行政执法事项50%。建立并公开政府部门公共服务目录，清理规范行政审批中介服务事项，服务功能不断优化。

　　深化重点领域改革。创新改革发展环境，获批东北地区民营经济发展改革示范市。组建了国资经营、交通运输、建设投资3个企业集团，完成2户企业国有股退出，国有资本布局日趋合理。妥善解决企业改革历史遗留问题，央企“三供一业”分离移交工作走在全省前列。化解政府债务56亿元，债务风险控制在合理区间。促进国税、地税服务深度融合，在全省率先实行“一窗一人双机双系统”联合办税模式。完成两县（市）中医院综合改革，实现基本药物制度基层医疗卫生机构全覆盖。完成不动产登记管理体制改革，率先实现“停旧发新”。深入推进“五证合一”，新增登记市场主体1.6万户。完成党政机关公车改革，年节约支出1300万元。

　　提高对外开放水平。实行重点出口企业包保责任制，严格执行奖惩机制。灵活运用代理出口方式，引进了2户外贸综合服务企业，培育了2户出口跨境电商平台。拓展出口领域，出口产品已达340余种、遍布100多个国家和地区。加强国际交流合作，主动融入“一带一路”倡议，3户企业实现境外投资，3户企业建立海外仓，成功开通中满欧果蔬冷链班列。

　　（七）大力发展社会事业，着力增进民生福祉

　　全面完成20项重点民生实事任务，以实际行动兑现了向广大市民作出的郑重承诺。

　　大力开展就业援助，新增实名制就业2.1万人，城镇登记失业率3%，零就业家庭保持动态为零。搭建了“智慧人社”平台。新增五险扩面2.6万人，新农合参合率保持在100%，城乡居民医保财政补助标准年人均提高到420元。城市低保、农村低保、农村五保供养标准分别提高5%、10%、7%。改造农村危房991户。建立养老保险基金缺口分级共担机制，退休人员养老金月人均提高到2149元，实现按时足额发放。

　　全面发展各项社会事业。文圣区跻身全国义务教育发展基本均衡地区。教育基础设施建设进一步加强，“三通两平台”实现全覆盖。医疗卫生服务体系不断完善，建立医联体，启动“云医院”等智慧卫生项目，重大疾病防控成效显著。文化惠民工程扎实推进，“书香辽阳”建设取得成效，30个“一卡通”社区图书馆投入运营。群众性体育活动丰富多彩，竞技体育综合实力不断增强。加速创建国家历史文化名城，编制东京城、燕州城和冮官屯窑址保护规划，启动白塔、台子沟等历史文化街区建设，完成关帝庙景区修缮。创建全国文明城市工作获省检第2名。国防教育和国防动员建设不断加强，“九一八”事变密谋地警示馆对外开放。气象、防震减灾、人防、地方志、档案、人口和计划生育、老龄、慈善、红十字和妇女儿童等事业都取得新进步。

　　加强社会管理综合治理。创新完善立体化社会治安防控体系，荣获全国公共安全视频监控联网应用示范城市。全面落实“打防管控服”各项措施，严厉打击黑恶势力，刑事发案稳中有降，多发性侵财案件和公共安全事故得到有效防范。深入开展社会矛盾纠纷排查调解专项行动，化解了一批信访积案。落实“党政同责、一岗双责”，安全生产形势保持平稳。强化食品药品监管，着力保障群众饮食用药安全。国家安全、民族宗教、外事侨务、涉台事务、应急管理、社区建设等工作取得新成效。连续第八次荣获全国双拥模范城称号。

　　（八）全面加强民主法治建设，进一步提高依法行政能力

　　主动接受市人大及其常委会的法律监督、工作监督，认真执行市人大及其常委会的决议和决定。坚持重大事项决策事前报告制度，积极回应人大代表对社会热点问题的关注。办理人大代表议案和建议174件。自觉接受市政协的民主监督，积极与政协开展民主协商，支持政协参政议政。办理政协提案249件。认真听取各民主党派、工商联、无党派人士、人民团体的意见和建议，积极支持工会、共青团、妇联等依照各自章程开展工作。

　　扎实开展“两学一做”学习教育，政风行风建设持续加强，机关服务能力和行政效率不断提高。深入落实党风廉政建设责任制，加大行政监察和审计监督力度，全面加强惩防体系建设。健全完善实绩考核机制，严格奖惩问责。启动“软环境建设年”活动，开展“三难”“三乱”等4个专项整治行动，发展环境进一步优化。

　　坚持依法行政。出台了《辽阳市人民政府拟定地方性法规草案和制定政府规章程序规定》，提请市人大常委会审议了《辽阳市城乡规划条例》。全面规范了行政处罚自由裁量权。深入开展法制宣传教育，法律服务能力不断增强。

　　各位代表，成绩的取得来之不易，是全市人民团结奋斗的结果；是市人大法律监督、工作监督和市政协民主监督、参政议政的结果；是驻军部队，中央、省驻我市单位，各民主党派、人民团体、社会各界人士、外来投资者和建设者关心支持的结果。在此，我代表市政府，向参与辽阳经济社会建设的全市人民和各界人士表示衷心的感谢！

　　在总结成绩的同时，我们也清醒地看到经济社会发展及工作中存在的问题和不足。一是受宏观经济环境和消化历史遗留问题等多重因素影响，部分经济指标增速回落较大，一些行业和企业生产经营还存在诸多困难。二是经济增长的传统动力减弱，新动能还没有形成支撑力量，企业创新能力和核心竞争力不强，产业结构深度调整任务艰巨。三是体制机制性矛盾尚未根本解决，改革创新的系统性、整体性、协同性有待加强，政府债务、养老金发放、社会风险管控等领域仍然存在一定隐患。四是政府自身建设还存在薄弱环节，少数干部不敢担当、不愿作为，“门好进、脸好看、事不办”的现象仍有发生。对于这些问题，我们要采取有力措施，认真加以解决。

　　二、2017年重点工作安排

　　2017年，是实施“十三五”规划的重要一年，是推进供给侧结构性改革的深化之年，也是“四个辽阳”建设的突破之年。尽管当前经济下行压力依然存在，但困难挑战中也蕴含着机遇和希望。我们看到，党中央、国务院坚定不移地实施新一轮东北振兴战略，政策举措效应必将进一步释放。我们看到，辽阳发展已形成明晰思路，市委、市政府全面推进“四个辽阳”建设，一批稳增长、调结构项目加快孕育，新的发展动能逐步形成。我们看到，省委不断修复政治生态，广大干部群众干事创业的动力在凝聚，加快发展的积极因素在涌现。只要我们坚定信心，抢抓机遇，乘势而上，就一定能够战胜前进道路上的艰难险阻，开创“四个辽阳”建设和振兴发展新局面。

　　今年政府工作的总体思路是：全面贯彻党的十八大和十八届三中、四中、五中、六中全会精神，深入学习贯彻习近平总书记系列重要讲话精神，按照中央、省委经济工作会议总体部署，紧紧围绕市十二次党代会和市委十二届三次全会确定的目标任务，坚持稳中求进工作总基调，统筹推进“五位一体”总体布局，协调推进“四个全面”战略布局，全面落实“五大发展理念”和“四个着力”，抢抓国家深入实施东北新一轮振兴战略的发展机遇，适应把握引领经济发展新常态，加快推进供给侧结构性改革，全面做好稳增长、促改革、调结构、惠民生、防风险各项工作，努力促进经济持续平稳健康发展、社会事业繁荣进步、民生福祉不断改善，为全面建成小康社会、扎实推进新一轮振兴发展、建设智慧文明美丽幸福新辽阳而努力奋斗。

　　全市经济发展的主要预期目标是：地区生产总值增长6.5%，一般公共预算收入增长5%，固定资产投资增长12%，城乡常住居民人均可支配收入均增长6.5%。

　　为实现上述目标，我们要重点抓好以下九项工作。

　　（一）不断完善体制机制，增强“四个辽阳”建设动力

　　切实优化投资营商环境。从转变政府职能入手，推动制度创新。开展优化投资营商环境专项行动，保护各类市场主体合法权益。推进东北地区民营经济发展改革示范市建设，努力营造开放包容、互利合作、重信守诺、亲商清商、尊商护商的浓厚氛围。

　　深入推进“放管服”改革。强力推进简政放权，继续取消下放行政审批事项，及时调整完善权责清单。各县（市）区成立行政审批局，完成相对集中行政审批权全域化改革。完善事中事后监管制度，“双随机、一公开”实现全覆盖。大力推进“互联网+政务服务”，简化办事流程，提高服务效率，最大程度方便群众和企业。

　　深化重点领域改革。启动厂办大集体改革，基本完成“三供一业”分离移交工作。组建旅游、科创、商贸企业集团。推进投融资体制改革，探索建立企业投资项目承诺制，积极开展政府和社会资本合作。推动国有企业混合所有制改革。启动城市公立医院改革，加快实施分级诊疗和基本药物制度。完善政府预算体系，落实结构性减税政策。加强政府债务管理，严格规范财政收支，年内化解政府债务22亿元。加速商事制度改革，广泛应用“一照一码”。推进事业单位分类改革和养老保险制度改革。完成事业单位公车改革。抓好供销社、盐业管理、国地税征管体制等领域改革。

　　努力扩大开放。用好用足政策，加快建设4个出口基地。充分发挥出口代理作用，为企业提供专业综合服务。完善佟二堡保税仓库功能。建立跨境电子商务平台，积极对接辽宁自贸区，推动更多产品打入国际市场。主动融入“一带一路”倡议，支持企业承包境外工程，推动产能合作。

　　发挥有效投资的关键作用。继续开展“项目年”活动，实施一批结构调整、基础设施和民生工程重点项目。积极争取中央7号文件、国务院28号和62号文件政策支持。大力推进招商引资，突出产业链招商、园区招商、全员招商、以商招商，实行项目全程服务，促进一批项目落地建设。

　　（二）深入实施“制造强市”战略，加速调整工业结构

　　全力帮扶实体经济。继续开展领导干部帮扶企业活动。引导和支持企业按法治化、市场化原则，积极稳妥降低杠杆率。推进债务源头企业进行债务清理整合，开展中小企业应收账款融资。推动新三板挂牌企业实现股权和转板融资。认真落实减税降费等政策，合理减轻企业负担。做强政府担保机构，拓展“助保贷”和“过桥”资金支持范围，有效降低企业融资成本。帮助企业争取直供电指标，完善土地供应制度，盘活利用闲置厂房10万平方米以上，努力降低企业用能用地成本。推动民营企业“大做强”“小升规”“个转企”。

　　加快壮大优势产业。全力支持辽化公司、忠旺集团等龙头企业发展，着力打造芳烃及精细化工、铝合金精深加工两个产值超千亿元产业基地。实施先进装备制造业升级工程，做优工程机械、汽车零部件等产业。

　　培育发展新兴产业。落实国家新兴产业发展三年行动计划，实施一批新材料、新能源汽车核心部件和节能环保等产业重点项目。支持电子信息产业园建设铜材产业基地。新兴产业主营业务收入占规上工业比重达到17%以上。

　　改造提升传统产业。巩固钢铁、煤炭去产能工作成果，坚决防止死灰复燃。按照“转型提质增效”的原则，引导钢铁企业发展科技含量高、附加值高的产品。支持企业兼并重组，有序退出一批水泥粉磨站和采选矿企业。实施消费品工业“三品”工程，推动袜业、童装等产业做大做强。

　　大力发展智能制造。积极探索“互联网+工业”发展新路径，推进智能化应用，促进信息化与工业化深度融合。制定扶持政策，扩大专项基金规模。建设5家自动化、数字化车间，培育2户省级以上试点示范企业，组织10户以上企业实施“机器换人”计划，助推工业结构优化升级。

　　（三）大力发展现代服务业，积极培育新的增长点

　　加快服务业集聚区建设。推进白塔中央商贸区提质升级，积极发展楼宇经济和总部经济。深入实施佟二堡国家级建制镇示范试点，加速区域产业发展。完善弓长岭温泉体验、休闲度假产业链条，打造全时全域旅游目的地。推进站西商务区建设，加速专业市场集聚。支持宏伟区发展工业物流和研发设计业。促进河东新城重点服务业项目建设和投入运营。

　　提升生产性服务业发展水平。大力发展金融业，支持城商行、农商行和其他金融机构拓展业务、发展壮大，力争再引进3家外埠金融机构。培育壮大3个电商基地，引导电商企业和实体企业对接，实现线上线下融合发展。提高乡村电商网点覆盖率，推动电子商务向农村发展。科学规划和布局物流节点，加快物流园区建设，积极推进与营口港合作发展“内陆港”。完善农产品现代流通体系和肉菜流通追溯体系，建设万隆农产品公益性批发市场。

　　加速生活性服务业转型升级。突出发展旅游业，围绕“一日游、两日游”目标人群，深度开发特色旅游资源。规划建设房车营地，实施“后备箱”工程。支持弓长岭、佟二堡创建国家5A、4A级景区。大力发展文体产业，鼓励乡镇影院建设，举办国际、国内高水平体育赛事。全面放开养老市场，推进公办养老机构社会化运作，启动国家级医养结合试点城市建设。引导家政服务向专业化、网络化方向发展。拓展乡村综合性服务网点布局，提高居民生活便利化水平。挖掘和培育“老字号”传统品牌。大力推进连锁经营和特许加盟，实现规模化和标准化经营。积极发展“夜经济”和会展经济，加快餐饮、酒吧等特色街区建设，推动生活性服务业扩总量、提质量。

　　（四）深入推进农业供给侧结构性改革，加速农业现代化进程

　　提高农业综合效益和竞争力。进一步提升9个示范区（带）建设水平，每个乡镇至少建成1个精品项目。调减玉米种植面积，培育3个优质粮食生产基地，粮食产量稳定在100万吨左右。完成农村土地承包经营权确权登记颁证“整市推进”任务。加快土地流转，大力发展适度规模经营，积极培育农村新型经营主体。开展化肥、农药减量增效试点，提高耕种收机械化水平。大力推进农业产业化，发展一批规模以上农产品加工企业，打造佟二堡皮草皮装省级农产品加工示范聚集区。深化辽阳县农村土地承包经营权抵押贷款试点，努力增加农民贷款。全面推进灯塔市国家级一二三产业融合发展试点建设，申报国家级可持续发展试验示范区。

　　完善农业农村基础设施。围绕提高粮食和重要农产品产能，大力实施“藏粮于地”战略，加快高标准农田建设，完成灯塔市万亩水稻示范试点任务。对太子河上游无堤段进行确权划界，启动管护工程建设。实施河道治理和农业灌溉工程，确保灌溉和防汛安全。再解决一批农村安全饮水问题，力争两年内实现全覆盖。大中修、改扩建各级公路526公里。

　　深入开展精准脱贫攻坚。全面实施产业、医疗、教育、住房、低保兜底救助等系列扶贫工程，确保1万人稳定脱贫和20个贫困村退出销号。

　　加快特色小镇建设。加大财政投入，创新融资方式，大力推进11个市级特色小镇基础设施和主导产业重点项目建设，促进产业、文化和旅游融合发展。推动佟二堡争创国家级特色小镇，再培育一批省级特色乡镇，建设升级版美丽乡村示范村108个。

　　（五）鼓励创新创业，激发“四个辽阳”建设活力

　　培育创新主体。深化产学研合作，推动企业与大连理工、中科院等科研院所无缝对接，提高企业科研实力。深入实施“双百”工程，大力发展高新技术企业，实现数量质量“双提升”。

　　推动技术创新。加强产业技术创新平台建设，新增5个省级技术中心和联合研发实验室。推进产业战略联盟和行业协会、商会建设。支持关键技术攻关和新产品开发，实施一批重大协同创新项目。

　　促进成果转化。丰富“成果转化项目库”，搭建科技成果转化综合服务平台。争取省产业直接投资基金，发挥2个海外研发中心科技优势，全力加速成果转化。鼓励企业引进先进技术，开展海内外并购。

　　打造创新高地。加快辽阳创新创业产业园区和中欧产业园建设，支持高新区、经济开发区体制机制创新，形成新的经济增长极。

　　构建创新环境。多渠道增加科技创新投入，营造鼓励创新创业的政策和制度环境。设立创业引导资金，加快建设中小微企业创业孵化基地和服务平台，推动大众创业、万众创新。加强知识产权保护和运用。

　　培养创新人才。推进市属高校、职业技术学校与重点企业进行对接，打造一批“辽阳工匠”。积极引进高端人才和海外研发团队，实施“十百千高端人才引进工程”，开展百名科技特派员进企业活动，不断提升创新创业水平。

　　（六）坚持建管并重，打造宜居城市

　　认真落实《辽阳市城乡规划条例》，严格执行《城市总体规划》，高标准编制重点区域城市设计和控制性详细规划，引领和提升城市建设水平。

　　推动新老城区融合发展。稳步建设河东新城，完善基础设施，推进太子河历史文化风光带和太子岛建设，丰富商业业态，不断聚集人气。加快老城区改建步伐，实施棚改项目38个、惠及居民1.1万户，推进东辽阳城区老工业区搬迁改造，综合改造老旧小区20个，继续实施绿化、美化、亮化工程，提升城市形象。

　　加强城市基础设施建设。实施大型人防公交枢纽工程，建成河东交通综合枢纽。开工建设西环路和西部路网，确保北环路竣工通车，进一步拓展城市骨架。完成新运大街电网落地工程，打通2个断头路，维修35条街巷道路，畅通群众出行。改造燃气、供水、供电、供热、环卫等基础设施，提高城市保障能力。

　　促进房地产业平稳健康发展。支持居民自住和改善性住房需求，建立购租并举、市场配置与政府保障相结合的住房制度，推动房地产回归居住属性。鼓励农民进城购房，全面推行居住证制度，落实并完善农业转移人口市民化的各项财政政策。筹办春秋两季房交会，拉动房地产销售。结合棚户区改造，有计划安排土地供应，促进住房供需基本平衡。

　　提升城市管理水平。整合城市管理综合执法队伍，集中行使住房城乡建设领域行政处罚权，推动城市管理走向城市治理。深入整治交通秩序，渠化改造8个道路交叉口，运用市场化方式建设停车场，进一步缓解“行车难、停车难”问题。开展环境卫生、集贸市场等综合整治，努力改善城市面貌。

　　（七）切实优化生态环境，推动绿色可持续发展

　　创建国家森林城市。封山育林和人工造林2.5万亩，森林覆盖率达到36.7%。实行严格的林地保护制度，严防“小开荒”反弹。加快推进湿地和自然保护区建设，争创太子岛国家级湿地公园，启动大黑山国家森林公园申报工作。

　　提升水体污染防治水平。全面推行河长制，实现“首长负责、部门共治”。深入贯彻国家“水十条”，加强饮用水源地环境保护，实施十大重点行业及工业水污染治理工程，提高全流域水环境质量。杜绝重金属污染和超标排放，城区污水处理厂排放均达到一级A标准。

　　巩固大气污染治理成果。坚决打胜治霾攻坚战，确保完成大气“国十条”确定的各项任务，空气质量优良天数持续增加。全面淘汰高污染燃料禁燃区内的燃煤锅炉，安装节能环保炉具1万台，更换新能源公交车100台。深入实施秸秆禁烧，推进秸秆综合利用。

　　开展土壤污染防治工作。积极落实“土十条”，编制《土壤污染防治工作方案》，加强污染状况排查，完成土壤环境质量国控监测点位设置和监测网络建设。（八）全力保障和改善民生，共享“四个辽阳”建设成果

　　继续稳定和扩大就业，支持高校毕业生和返乡农民工创业就业，城镇新增就业1.6万人，城镇登记失业率控制在3.5%以内。依法推进社保扩面征缴，实施全民参保登记计划。积极化解养老金风险，确保按时足额发放。进一步提高城乡低保标准。分配公租房1200套，发放廉租房补贴3500户。提前彻底消除1760户农村C、D级危房。

　　促进各级各类教育均衡发展。推进协作化办学，广泛应用“三通两平台”。加快校园基础设施建设，改造义务教育薄弱学校。积极引进高水平教师，强化师德师风建设。力争太子河区进入国家义务教育发展均衡地区，全市学前毛入园率达到90%，高考重点院校入学率提高3个百分点。开展校园系列专项整治，确保校园安全。

　　完善医疗卫生服务体系。强化重大疾病防控和城乡居民健康管理。推进资源共享，实现云医院和医联体全覆盖。整合城乡居民基本医保，合并实施生育保险和基本医疗保险，加快实现医保异地就医结算。新农合参合率保持在100%。

　　积极发展文化体育事业。完善公共文化体系，加强基层综合文化站和文化中心建设。保护明城墙遗址，建设白塔、台子沟等历史文化街区，加速创建国家历史文化名城。适时启动东京城保护利用和魁星楼复建工程，积极推进燕州城和冮官窑遗址公园建设。丰富群众文化生活，建设2个自助图书馆，河东大剧院投入运营。大力发展竞技体育，办好市第七届全民运动会，推动国有体育场馆公司化运营。

　　加强和创新社会治理。加快完善立体化治安防控体系，深入推进常态化打击整治，加大民生案件侦破力度，不断增强人民群众安全感。集中开展“信访积案化解年”活动，努力削减信访存量，减少社会不稳定因素。强化生产、消防、交通、食品药品等安全监管，提高突发事件应急处置和防灾减灾能力。加强全民国防教育和国防动员建设，继续争创全国双拥模范城。搭建公共信息资源共享交换平台，全面推进信息惠民工程建设。创新社区和社会组织管理模式。加速创建全国文明城市，开展国家卫生城市创建工作。

　　（九）强化政府自身建设，努力提升执政能力和水平

　　主动接受市人大及其常委会的法律监督和工作监督，自觉接受市政协的民主监督。及时向市人大及其常委会报告工作，认真执行市人大及其常委会的决议、决定和审议意见，认真办理人大代表的议案、建议。积极支持政协参政议政，提高政协提案办理质量，充分听取政协以及各民主党派、工商联、无党派人士、人民团体的意见和建议。

　　建设法治政府。实施《辽阳市法治政府建设方案（2016-2020）》，依法履行政府职能。认真落实《立法法》，加强立法工作。优化法治环境，规范行政执法行为。全面启动“七五”普法。

　　建设诚信政府。严格履行政府各项承诺，做到言必信、行必果，切实增强政府公信力。扎实推进国家社会信用体系示范市建设，营造“守信受益、失信受损”的社会氛围。

　　建设高效政府。狠抓工作落实，将省委、省政府和市委的顶层设计、决策部署转化为具体操作方案，制定时间表、路线图，努力提高工作效率。

　　建设服务政府。牢固树立执政为民理念，切实增强主动服务意识。坚决纠正不作为、乱作为问题，积极倡导真抓实干、勇于担当的工作作风。

　　建设廉洁政府。认真落实党风廉政建设责任制，加强行政监察和审计监督，强化惩防体系建设，用制度管住权力、预防腐败，营造良好的政治生态。

　　各位代表，民生连着民心，民心凝聚民力。我们要谋民生之利，解民生之忧，在全面做好各项民生工作的基础上，着力办好20项重点民生实事，让群众共享发展成果。

　　各位代表，宏伟的事业激励着我们，人民的期待鞭策着我们，美好的前景鼓舞着我们。让我们紧密团结在以习近平同志为核心的党中央周围，在省委、省政府和市委的正确领导下，咬定青山不放松，面对困难不退缩，团结拼搏不懈怠，励精图治，开拓创新，扎实推进新一轮振兴发展，努力建设智慧文明美丽幸福新辽阳，以优异成绩迎接党的十九大胜利召开！
